# Research for Expression and Prognostic Value of GABRD in Colon Cancer and Coexpressed Gene Network Construction Based on Data Mining

**DOI:** 10.1155/2021/5544182

**Published:** 2021-06-07

**Authors:** Tao Liu, Yuejun Fang

**Affiliations:** Department of Gastrointestinal Surgery, Zhejiang Jinhua Guangfu Tumor Hospital, Jinhua 321000, China

## Abstract

Colon cancer is one of the top five cancers with the highest incidence rate in the world. In order to better understand the pathogenesis and progression of colon cancer, it is still necessary to investigate the abnormally expressed genes in cancer tissue. In this study, the Oncomine database was used for expression analysis, and it was found that the expression level of gamma-aminobutyric acid type A receptor subunit delta (GABRD) gene was upregulated in colon cancer tissue compared with that in normal tissue. UALCAN was used to analyze the expression of GABRD in different groups of age, gender, cancer stage, N stage, and histological subtype. Then, it was also found that the expression of GABRD in each subgroup of colon cancer tissue was all high compared with that in normal tissue. LinkedOmics was used to screen out the differentially expressed genes related to GABRD expression in colon cancer. GO annotation and KEGG pathway enrichment analyses found that the correlated genes may be related to breast cancer, human papillomavirus infection, Notch signaling pathway, and other pathways. Thereafter, GSEA was performed to obtain GABRD-related kinases, miRNAs, and transcription factors, and gene interaction networks were constructed. It was found that GABRD may be involved in cell cycle regulation. Finally, websites like GEPIA were used to detect the predictive ability of GABRD on the prognosis of patients with colon cancer. Kaplan-Meier analysis suggested that the upregulation of GABRD expression was related to the poor prognosis of patients with colon cancer. Overall, in this study, the potential role and prognostic ability of GABRD in colon cancer were explored through data mining, which can be a clue for further research on GABRD.

## 1. Introduction

Colon cancer is a very common cancer. Its incidence rate is the fourth next to lung cancer, breast cancer, and prostate cancer in the world, with nearly 6% of cancer deaths caused by colon cancer each year [1]. The 5-year survival rate of colon cancer is about 60%. Although the mortality rate in developed countries is declining due to the popularization of colon cancer screening, the number of patients under the age of 50 is increasing, and colon cancer in young patients is often more aggressive [2]. Moreover, the incidence rate of colon cancer in developing countries is demonstrating an upward trend with the development of the economy, which is considered to be related to changes in eating habits [3, 4]. It can be seen that the overall situation of colon cancer in the world is still relatively severe, and further research on the molecular regulatory mechanism of its progression is needed to support the development of drugs and diagnostic markers.

GABAAR, one of the two types of gamma-aminobutyric acid A (GABAA) receptor, is a member of the Cys-loop family of pentameric transmembrane ligand-gated ion channels. It is expressed in the nervous system as well as related to the advanced functions of the human brain; thus, there are multiple neural-related studies [5–7]. Gamma-aminobutyric acid type A receptor subunit delta (GABRD) as one of the subunits of GABAAR is found to be closely related to cancer. A study of pan-cancer analysis found that GABRD is one of the most upregulated genes in tumor tissue [8]. The analysis for hepatocellular carcinoma found that GABRD is remarkably upregulated in each cancer stage [9]. However, in low-grade gliomas, there are reports claiming that GABRD can be a prognostic marker, and patients with low GABRD expression often have a poor prognosis [10, 11]. It can be seen that the functional mechanism of GABRD in cancer needs to be studied on specific cancer types.

At present, there are still very few studies on GABRD in cancer. However, there were studies which constructed predictive models for prognostic risk using data of colon cancer patients and found that lowly expressed GABRD is remarkably associated with good overall survival (OS) [12, 13]. Also, a report claimed that the high expression of GABRD mRNA can promote the progression of colon cancer to advanced TNM stages [14]. Therefore, this study chose to explore GABRD in colon cancer. The data of colon cancer patients from The Cancer Genome Atlas (TCGA), CPTAC, and other datasets were used to analyze the expression of GABRD. The potential regulatory mechanism in cancer was then explored, thus offering a deeper understanding of the relationship between GABRD and colon cancer. This also provides support for GABRD to become a prognostic marker for colon cancer patients, as well as offers clues for further research on relevant functional mechanisms.

## 2. Materials and Methods

### 2.1. Analysis of GABRD Expression in Colon Cancer Patients

Colon cancer datasets Zou Colon, Gaspar Colon, and Skrzypczak Colorectal in Oncomine database (https://www.oncomine.org/resource/login.html) [15] were used to conduct a comparative analysis of GABRD mRNA expression in cancer tissue and normal tissue. Fold change of GABRD expression was obtained, which represented the mean value of the expression in cancer tissue divided by the mean value of its expression in normal tissue. At the same time, the samples of colon cancer patients in the TCGA-COAD dataset were divided into subgroups according to age, gender, histological type, N stage, cancer stage, and sample types (normal tissue and tumor tissue). UALCAN (http://ualcan.path.uab.edu/index.html) was used to analyze GABRD expression [16]. A *T* test was performed to detect whether the difference in GABRD expression in cancer tissue was not able compared with that in normal tissue. *p* < 0.05 represents that the difference is significant.

### 2.2. Mining of Coexpressed Genes of GABRD in Colon Cancer

Analysis in the CPTAC-COAD dataset was conducted using the *LinkFinder* module of LinkedOmics (http://www.linkedomics.org/) [17] to obtain the differentially expressed genes associated with GABRD expression, which was demonstrated in a volcano plot. The *LinkFinder* module used Pearson correlation analysis to detect the correlation between each gene and GABRD in the dataset, with *p* < 0.05 as the screening threshold. Meanwhile, a heat map of the top 50 genes with the most significant positive or negative correlation with GABRD was obtained. Thereafter, genes with ∣Pearson correlation coefficient | >0.5 and *p* < 0.05 were defined as the coexpressed genes of GABRD.

### 2.3. Gene Ontology (GO) Annotation and Kyoto Encyclopedia of Genes and Genomes (KEGG) Pathway Enrichment Analysis

(GO) annotation and KEGG pathway enrichment analyses were performed for the coexpressed genes of GABRD screened by LinkedOmics using R package *clusterProfiler* [18], and the screening threshold was FDR (False Discovery Rate) < 0.05. GO annotation was implemented to obtain results from the following three dimensions: biological process (BP), cellular component (CC), and molecular function (MF).

### 2.4. Enrichment Analysis of Kinase Targets, miRNA Targets, and Transcription Factor Targets and Construction of Interaction Networks

The Gene Set Enrichment Analysis (GSEA) tool of LinkedOmics was used to conduct enrichment analysis on kinase targets, miRNA targets, and transcription factor targets with the data obtained by *LinkFinder* analysis. FDR was selected as the rank criterion, and the simulation times were set to 500. Based on the results of the enrichment analysis, the gene sets with the lowest FDR were chosen. GeneMANIA (https://genemania.org/search/homo-sapiens/) [19]was used to construct gene interaction networks. Correlations between genes were analyzed (physical interaction, coexpression, predicted, pathway, colocation, and shared protein domain), and the function of genes in networks was predicted.

### 2.5. Prognostic Analysis

Gene Expression Profiling Interactive Analysis (GEPIA) (http://gepia.cancer-pku.cn/detail.php), Tumor IMmune Estimation Resource (TIMER) (https://cistrome.shinyapps.io/timer/), and UALCAN [20, 21] were employed to detect the potential prognostic ability of GABRD. On the GEPIA website, the upper and lower quartiles of GABRD expression in all samples of TCGA-COAD were taken as dividing lines, and patients were then divided into the high-expression group (expression was higher than the upper quartile) and the low-expression group (expression was lower than the lower quartile). On the TIMER website, TCGA-COAD patients were distinguished by the median value of GABRD expression. On the UALCAN website, TCGA-COAD patients with higher GABRD expression than the third quartile were classified as the high-expression group, while the rest were classified as the low-expression group. Kaplan-Meier (KM) analysis was performed on the data of the two groups. *p* < 0.05 indicates a notable difference.

## 3. Results

### 3.1. GABRD Is Highly Expressed in Colon Cancer Patients

**Figure 1:**
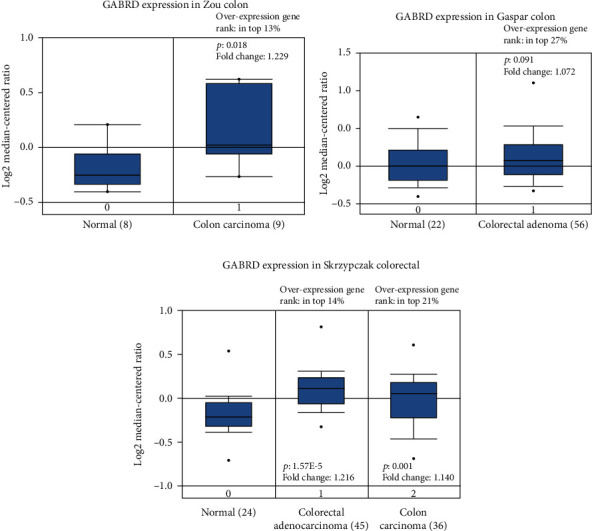
GABRD is highly expressed in colon cancer. (a–c) Boxplots of GABRD mRNA expression in tumor tissue and normal tissue of colorectal cancer patients in the Zou Colon dataset (a), Gaspar Colon dataset (b), and Skrzypczak Colorectal dataset (c) in the Oncomine database. The overexpression gene rank is the percentile rank of the significance of GABRD overexpression in all overexpressed genes in the datasets. The greater the significance, the higher the rank.

**Figure 2:**
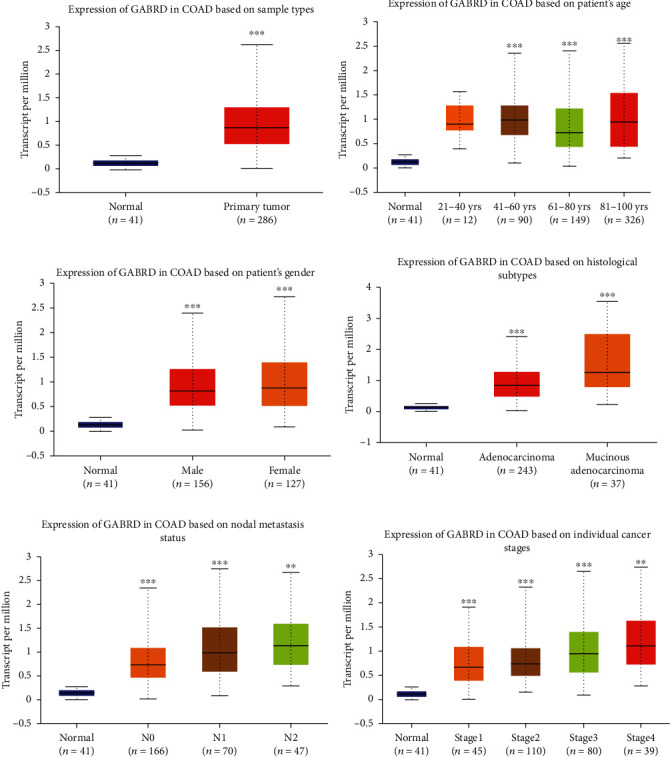
In the TCGA-COAD dataset, GABRD expression is upregulated in colon cancer patients from different subgroups of age, gender, etc. (a) GABRD expression in normal tissue and colon cancer tissue; (b) GABRD expression in normal tissue and colon cancer tissue of the patients from four age subgroups (21-40 yrs, 41-60 yrs, 61-80 yrs, and 81-100 yrs); (c) GABRD expression in normal tissue and colon cancer tissue of the patients from two gender subgroups (male and female); (d) GABRD expression in normal tissue and colon cancer tissue of the patients from two histological subgroups (adenocarcinoma and mucinous adenocarcinoma); (e) GABRD expression in normal tissue and colon cancer tissue of the patients from three N staging subgroups (N0, N1, and N2); (f) GABRD expression in normal tissue and colon cancer tissue of the patients from four subgroups of cancer stages (stages 1, 2, 3, and 4). ∗∗ represents *p* < 0.01; ∗∗∗ represents *p* < 0.001.

We analyzed multiple colorectal cancer datasets with the Oncomine database and found that GABRD was prominently overexpressed in most of the cancer tissue in the datasets (*p* < 0.05). The significance of overexpression of GABRD mRNA was in the top 30% of overexpressed genes in these datasets (Figures 1(a)–1(c)). In order to further investigate the changes in GABRD expression in colon cancer, UALCAN was applied to analyze the colon cancer patients in the TCGA-COAD dataset, and the result also suggested that GABRD expression in cancer tissue was high compared with that in normal tissue (Figure 2(a)). The colon cancer patients were divided into groups according to age, gender, and histological subtype, and then, it was revealed that compared with the normal group, GABRD expression was remarkably upregulated in each subgroup except the 21-40 yrs subgroup (Figures 2(b)–2(d)). At the same time, after the patients were divided according to the N stage and cancer stage, each subgroup exhibited an increasing trend toward GABRD expression with the progression of colon cancer (Figures 2(e) and 2(f)). It could be seen that GABRD may be associated with the occurrence and progression of colon cancer.

### 3.2. Screening of Coexpressed Genes of GABRD and Subsequent GO Annotation and KEGG Pathway Enrichment Analysis

**Figure 3:**
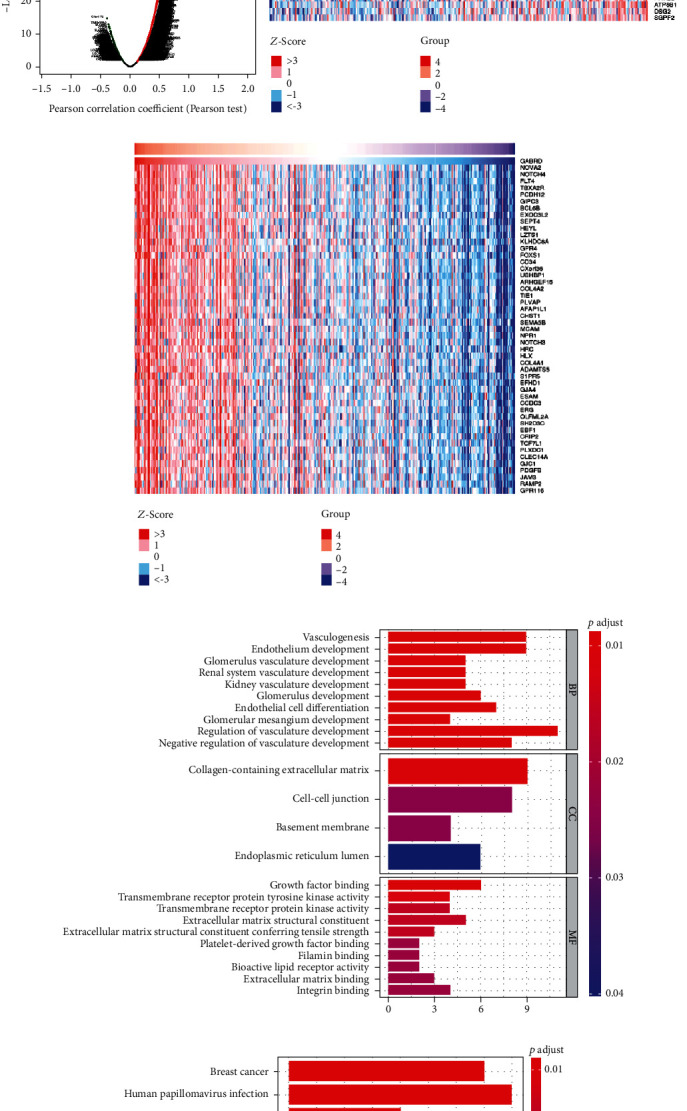
Genes related to GABRD expression in colon cancer. (a) Volcano plot of Pearson correlation of GABRD and the differentially expressed genes in the CPTAC-COAD dataset. The green dots represent the genes significantly negatively correlated with GABRD; the red dots represent the genes notably positively correlated with GABRD; the black dots are the genes not significantly correlated with GABRD; (b, c) expression of the genes remarkably positively correlated with GABRD and negatively correlated with GABRD demonstrated in two heat maps, respectively (top 50 genes were selected according to *p* value); (d) coexpressed genes of GABRD in colon cancer were analyzed by GO annotation; (e) KEGG pathway enrichment analysis was conducted on the coexpressed genes of GABRD.

In order to study the possible function of GABRD in colon cancer, *LinkFinder* of LinkedOmics was employed to analyze the gene expression data of colon cancer patients from the CPTAC-COAD dataset, and the genes associated with GABRD expression was extracted from the differentially expressed genes in cancer tissue (*p* < 0.05) (Figure 3(a)). Meanwhile, the top 50 genes with the most notable positive or negative correlation with GABRD were selected, respectively, and displayed in the form of a heat map (Figures 3(b) and 3(c)). Then, coexpressed genes of GABRD were screened from the obtained GABRD-correlated genes (∣Pearson correlation coefficient | >0.5, *p* < 0.05, Table S1), and a total of 79 coexpressed genes of GABRD were obtained. GO annotation and KEGG pathway enrichment analyses were conducted on these coexpressed genes (FDR < 0.05). The result of GO analysis indicated that the coexpressed genes of GABRD were notably involved in BP, such as vasculogenesis and endothelium development; in MF like growth factor binding and transmembrane receptor protein tyrosine kinase activity; and in CC, such as collagen-containing extracellular matrix (Figure 3(d)). The result of KEGG enrichment analysis suggested that the coexpressed genes of GABRD were significantly involved in breast cancer, human papillomavirus infection, Notch signaling pathway, and other pathways (Figure 3(e)).

### 3.3. Network Construction of GABRD-Related Kinase, miRNA, and Transcription Factor Targets in Colon Cancer

**Figure 4:**
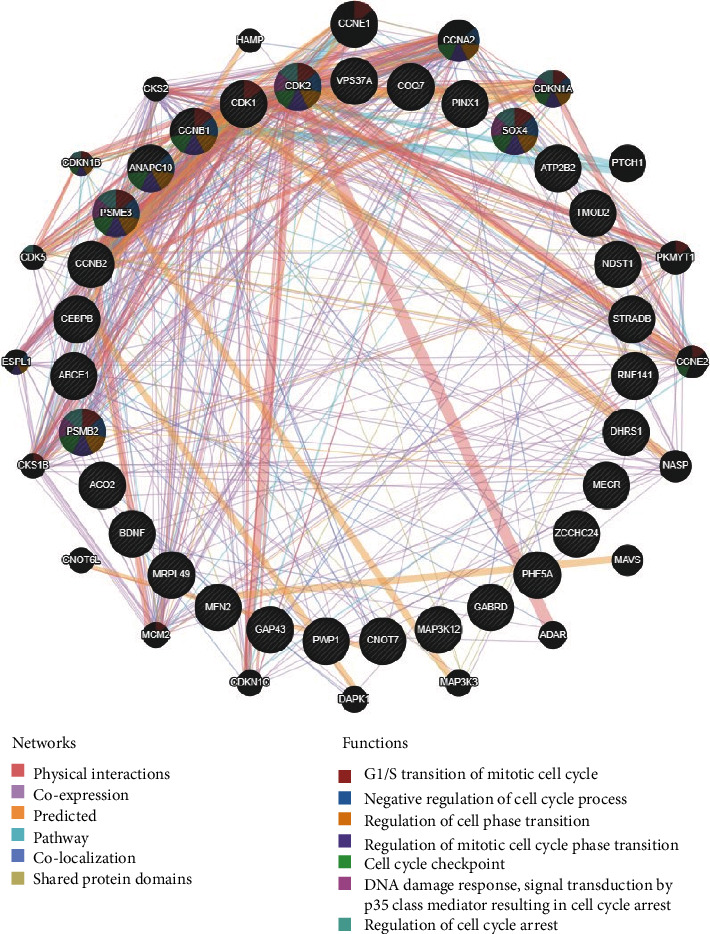
Gene interaction network of target kinases, miRNAs, and transcription factors of GABRD. The slashed circles represent the genes presented in GSEA analysis in the networks of WEE1 G2 checkpoint kinase, miR-191 (TTCCGTT), and GKCGCNNNNNTGAYG_UNKNOWN, and the pure black circles represent the interaction genes obtained by network construction. The different colored lines between circles indicate different interactions shown in the network such as physical interactions and coexpressions. The different colored circular sectors represent the biological functions of the gene set enrichment in the interaction network.

In order to further understand the possible role of GABRD in colon cancer, enrichment analysis was carried out on kinase, miRNA, and transcription factor targets of GABRD using the GSEA tool of LinkedOmics (FDR < 0.25), and target regulatory networks related to GABRD were mined. Among them, networks of kinase, miRNA, and transcription factor targets with the lowest FDR value were WEE1 G2 checkpoint kinase, miR-191 (TTCCGTT), and GKCGCNNNNNNNTGAYG_UNKNOWN, respectively (Table 1). It was indicated that GABRD was likely to be regulated by these networks or play a role in colon cancer through these target networks. In order to explore the function of the target networks obtained through GSEA enrichment analysis, GeneMANIA was used to construct gene interaction networks for the above three target networks. It was found that G1/S transition of mitotic cell cycle, negative regulation of cell cycle process, regulation of cell cycle phase transition, and other functions were notably enriched in the constructed networks (Figure 4). This manifested that GABRD-related kinase, miRNA, and transcription factor targets mainly played a role in cell cycle regulation, and GABRD may also affect the occurrence and progression of colon cancer through this regulatory function.

### 3.4. Detection of the Prognostic Potential of GABRD Gene in Colon Cancer Patients

**Figure 5:**
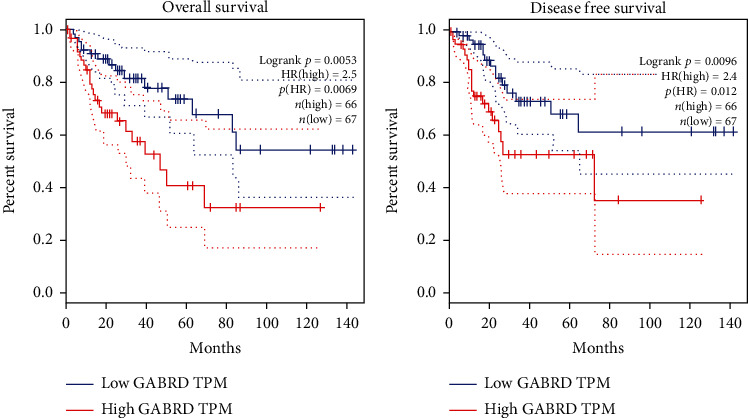
GABRD expression is positively correlated with the poor prognosis of colon cancer patients. (a) KM analysis was used to test the OS rate in the GABRD high-expression group and the low-expression group; (b) the DFS rate in the GABRD high-expression group and the low-expression group tested by KM analysis.

As abovementioned, GABRD expression elevated with the progression of colon cancer, and the coexpressed genes of GABRD were enriched in cancer progression-related biological functions and pathways. Given this, it was suggested that GABRD may be associated with the malignant progression of colon cancer. By using the GEPIA tool, KM analysis was conducted to detect the relationship between GABRD expression and prognosis of colon cancer patients, and the results manifested that the OS rate of patients in the GABRD low-expression group was significantly higher than that in the high-expression group (Figure 5(a)). Similarly, the DFS rate of patients in the GABRD low-expression group was notably higher than that in the high-expression group (Figure 5(b)). In addition, analysis on TIMER and UALCAN websites both revealed that COAD patients with low GABRD expression had a better prognosis (Figure S1A-B). It could be seen that the expression level of GABRD was negatively correlated with the good prognosis of colon cancer patients, and GABRD could be a potential marker of the prognosis of colon cancer.

## 4. Discussion

In this study, the LinkedOmics database was employed to analyze the coexpressed genes associated with GABRD expression, and the genes with high correlation coefficients were screened for enrichment analysis. The result of KEGG enrichment analysis suggested that the coexpressed genes with high correlation with GABRD in colon cancer were highly enriched in breast cancer and human papillomavirus (HPV) infection pathway, suggesting that GABRD may also play a regulatory part in breast cancer, which had not been studied in the past. At the same time, previous statistics revealed that a high proportion of patients with genital cancers like penile cancer and vaginal cancer as well as anal cancer are infected with high-risk HPV, and infection with high-risk HPV is considered a necessary cause for cervical cancer [22]. This suggests that GABRD and its coexpressed genes may be potentially associated with the above cancer types through the HPV infection pathway and also indicates the relationship between colon cancer and HPV.

In addition, coexpressed genes of GABRD also appeared with highly remarkable enrichment in the pathway involved in focal adhesion. Focal adhesion is a crucial form of cell adhesion, and it is composed of integrins that connect the extracellular matrix, intracellular signaling pathway, and cytoskeleton to detect the extracellular environment and respond to it [23]. Integrins are often abnormally expressed in cancer cells, and they are involved in regulating the migration and invasion of cancer cells through signal transduction by FAK (focal adhesion kinase) and Talin [24]. A study analyzed the molecular interaction network of transition from focal adhesions to invadopodia to explore the transition of noninvasive cells to invasive cells and identified that the upstream regulatory centers of this transition are PI3K and PKC*α*. In addition, the high activity of PI3K can promote the generation of invadopodia [25]. At the same time, in this study, the PI3K-Akt signaling pathway also appeared in the enrichment analysis results of GABRD coexpressed genes. Currently, it has been found that the functional mechanisms of various anticancer drugs or components are related to the PI3K-Akt signaling pathway. For example, resveratrol inhibits the PI3K-Akt signaling pathway by upregulating BMP7, thus promoting the apoptosis of colon cancer cells and suppressing the proliferation of colon cancer cells [26]. Coptisine can also promote the apoptosis of colon cancer cells by inhibiting the PI3K-Akt signaling pathway [27]. It can be seen that the PI3K-Akt signaling pathway plays a vital role in the regulation of proliferative and other abilities of colon cancer cells. Coexpressed genes of GABRD were also found to be highly enriched in the Notch signaling pathway. Activation of the Notch signaling pathway was found to be positively correlated with the generation and maintenance of stemness of cancer stem cells (CSCs) in colon cancer [28–30], and CSCs are considered to be the reason for cancer occurrence and difficult treatment [31]. These pathways where the coexpressed genes are highly enriched may have a great influence on the development of colon cancer, and GABRD may be regulated by these pathways or participate in the regulation of these pathways.

Then, in this research, the GSEA tool of LinkedOmics was applied to perform enrichment analysis on kinase targets, miRNA targets, and transcription factor targets, and the possible target networks of GABRD in colon cancer were obtained. A notable enrichment was presented in the miR-191 network when enrichment analysis was performed on miRNA targets. Previous research manifested that miR-191 can promote colorectal cancer tumor formation by suppressing C/EBP*β* expression [32]. Triptolide inhibits the migration and progression of colon cancer cells by downregulating miR-191 [33]. Meanwhile, enrichment of WEE1 kinase was most noticeable in kinase target networks. WEE1 plays a significant role in G2/M cell cycle checkpoint arrest, and the occurrence of G2/M phase arrest is conducive to DNA repair of cancer cells [34]. In colon cancer, sulforaphane and curcumin are reported to be able to promote G2/M phase arrest in colon cancer cells by upregulating WEE1 expression, which finally leads to the apoptosis of cancer cells [35, 36]. Previous studies showed that transcription factors can play a regulatory part in the progression of colon cancer [37]. However, no known transcription factor binding sites have been found to match the motif corresponding to the most significantly enriched gene set in this study. Here, the GeneMANIA network was constructed using genes enriched in the three target networks with the most notable enrichment. The results suggested that genes in the target networks exhibited remarkable enrichment in functions related to cell cycle, including G1/S transition of the mitotic cell cycle, negative regulation of cell cycle process, and regulation of cell cycle phase transition, and they are highly correlated with the function of WEE1 kinase. Currently, the development of drugs aimed at the cell cycle has become one of the pivotal directions in cancer treatment [38]. It can be seen that GABRD may participate in the regulation of the cell cycle through the WEE1 kinase network and other target networks; thus, GABRD may be a target for cancer therapy.

In general, in this research, by mining the colon cancer dataset in the Oncomine database, it was uncovered that GABRD expression was higher in colon cancer tissue compared with that in normal tissue. In addition, after the groups were divided according to age, gender, histological subtype, etc., GABRD expression levels in each subgroup were still notably higher than that in the normal group. Next, the biological functions of GABRD and its coexpressed genes were explored by LinkedOmics and GeneMANIA, and the prognostic potential of GABRD was tested. In all, our study lays a foundation for further research on GABRD in the future.

## Figures and Tables

**Table 1:** Networks of kinase, miRNA, and transcription factor targets of GABRD.

Networks	Gene set	Description	Size	Leading edge number	ES	NES	*p* value	FDR
Kinase target	Kinase_WEE1	WEE1 G2 checkpoint kinase	5	4	-0.92033	-1.7192	0.0070922	0.17746
	Kinase_NEK2	NIMA-related kinase 2	8	5	-0.74354	-1.746	0.021053	0.18368
	Kinase_PKM	Pyruvate kinase, muscle	4	2	-0.95009	-1.7604	≤0.001	0.235
	Kinase_CSNK1G2	Casein kinase 1 gamma 2	6	3	0.94765	1.5272	0.0026316	0.2712

miRNA target	TTCCGTT,MIR-191		29	9	0.64417	1.2699	0.10408	0.19167
	ATACCTC,MIR-202		166	56	0.60966	1.2719	0.002	0.19248
	ATGCTGG,MIR-338		104	23	0.62396	1.2758	0.006	0.19375
	ATGTAGC,MIR-221,MIR-222		133	45	0.62396	1.2766	0.004	0.19849
	AACATTC,MIR-409-3P		132	32	0.60846	1.2587	0.004008	0.19852

Transcription factor target	GKCGCNNNNNNNTGAYG_UNKNOWN		52	17	-0.52265	-2.0365	≤0.001	0
	V$CEBP_01		244	71	0.61884	1.2806	≤0.001	0.028315
	V$IRF1_01		229	72	0.62167	1.2947	≤0.001	0.028349
	V$STAT5A_01		231	77	0.62077	1.2809	≤0.001	0.028362
	V$TTF1_Q6		243	83	0.62774	1.2951	≤0.001	0.028446

## Data Availability

The data used to support the findings of this study are included within the article. The data and materials in the current study are available from the corresponding author on reasonable request.

## Supplementary Materials

Supplementary Materials Table S1: screening results of coexpressed genes of GABRD. Figure S1: survival analysis of the GABRD high- and low-expression groups based on TCGA-COAD patient data: (A) results of KM survival analysis on the TIMER website; (B) results of KM survival analysis on the UALCAN website. The red line represents the high-expression group while the blue line represents the low-expression group.
