# Supplementary material for: Research for Expression and Prognostic Value of GABRD in Colon Cancer and Coexpressed Gene Network Construction Based on Data Mining
Source: Comput Math Methods Med. 2021 Jun 7;2021:5544182. doi: 10.1155/2021/5544182 (PMC8203377; doi:10.1155/2021/5544182)
Supplement: Supplementary Materials — Table S1: screening results of coexpressed genes of GABRD. Figure S1: survival analysis of the GABRD high- and low-expression groups based on TCGA-COAD patient data: (A) results of KM survival analysis on the TIMER website; (B) results of KM survival analysis on the UALCAN website. The red line represents the high-expression group while the blue line represents the low-expression group. [file 5544182.f1.zip › Table S1.pdf]

NOTCH4
NOVA2
FLT4
PCDH12
EXOC3L2
TBXA2R
BCL6B
GIPC3
HEYL
SEPTIN4
LZTS1
USHBP1
CD34
TMEM233
HRC
FOXS1
COL4A2
GPR4
DIPK2B
MCAM
ADAMTS5
ARHGEF15
CHST1
NOTCH3
TIE1
KLHDC8A
EFHD1
AFAP1L1
GJC1
SEMA5B
PLVAP
NPR1
COL4A1
S1PR5
CCDC3
ESAM
HEY2
HLX
GJA4
OLFML2A
S1PR3
SLC6A1
ERG
TCF7L1
CSPG4
PDGFB
HECW2
FLT1
JAM3
EBF1
KDR
SOX18
CLEC14A
CRIP2
RBP7
EMCN
FZD4
ARHGEF17

ADGRF5
VASN
PLXDC1
MYCT1
LDB2
MGP
MMRN2
GPIHBP1
SH2D3C
ACTA2
RASIP1
NGF
COL18A1
TMEM204
SOX17
ROBO4
LHFPL6
SLC6A17
RFLNB
CRMP1
SYDE1
