# Supplementary figures and images for: Research for Expression and Prognostic Value of GABRD in Colon Cancer and Coexpressed Gene Network Construction Based on Data Mining
Source: Comput Math Methods Med. 2021 Jun 7;2021:5544182. doi: 10.1155/2021/5544182 (PMC8203377; doi:10.1155/2021/5544182)

Figure S1

A

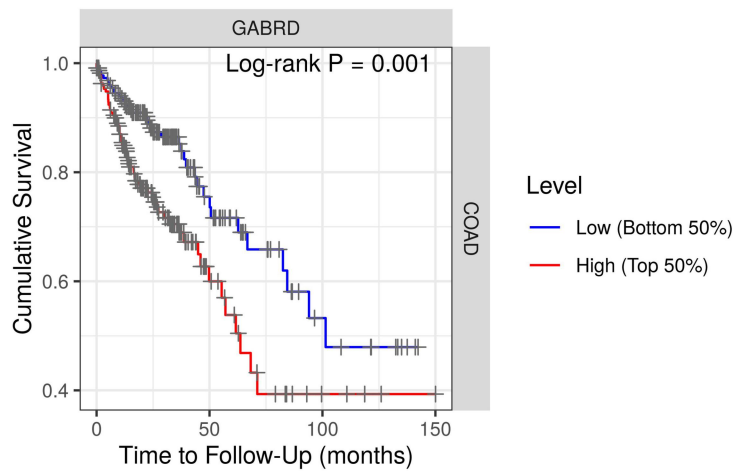

B

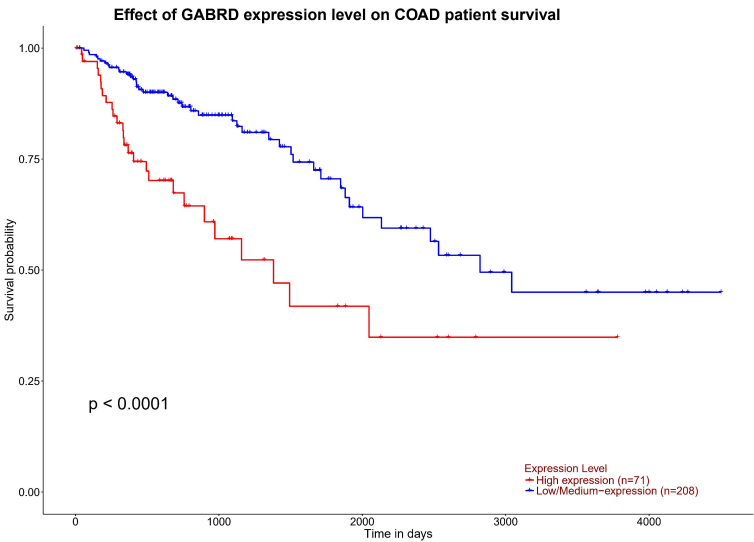

Supplement: Supplementary Materials — Table S1: screening results of coexpressed genes of GABRD. Figure S1: survival analysis of the GABRD high- and low-expression groups based on TCGA-COAD patient data: (A) results of KM survival analysis on the TIMER website; (B) results of KM survival analysis on the UALCAN website. The red line represents the high-expression group while the blue line represents the low-expression group. [file 5544182.f1.zip › Figure S1.pdf]
